# Measuring agreement of administrative data with chart data using prevalence unadjusted and adjusted kappa

**DOI:** 10.1186/1471-2288-9-5

**Published:** 2009-01-21

**Authors:** Guanmin Chen, Peter Faris, Brenda Hemmelgarn, Robin L Walker, Hude Quan

**Affiliations:** 1 Department of Community Health Sciences, University of Calgary, Calgary, Alberta, Canada; 2 Alberta Bone and Joint Health Institute, Calgary, Alberta, Canada; 3 Department of Medicine, University of Calgary, Calgary, Alberta, Canada; 4 Centre for Health and Policy Studies, University of Calgary, 3330 Hospital Dr. NW, Calgary, Alberta T2N 4N1, Canada

## Abstract

**Background:**

Kappa is commonly used when assessing the agreement of conditions with reference standard, but has been criticized for being highly dependent on the prevalence. To overcome this limitation, a prevalence-adjusted and bias-adjusted kappa (PABAK) has been developed. The purpose of this study is to demonstrate the performance of Kappa and PABAK, and assess the agreement between hospital discharge administrative data and chart review data conditions.

**Methods:**

The agreement was compared for random sampling, restricted sampling by conditions, and case-control sampling from the four teaching hospitals in Alberta, Canada from ICD10 administrative data during January 1, 2003 and June 30, 2003. A total of 4,008 hospital discharge records and chart view, linked for personal unique identifier and admission date, for 32 conditions of random sampling were analyzed. The restricted sample for hypertension, myocardial infarction and congestive heart failure, and case-control sample for those three conditions were extracted from random sample. The prevalence, kappa, PABAK, positive agreement, negative agreement for the condition was compared for each of three samples.

**Results:**

The prevalence of each condition was highly dependent on the sampling method, and this variation in prevalence had a significant effect on both kappa and PABAK. PABAK values were obviously high for certain conditions with low kappa values. The gap between these two statistical values for the same condition narrowed as the prevalence of the condition approached 50%.

**Conclusion:**

Kappa values varied more widely than PABAK values across the 32 conditions. PABAK values should usually not be interpreted as measuring the same agreement as kappa in administrative data, particular for the condition with low prevalence. There is no single statistic measuring agreement that captures the desired information for validity of administrative data. Researchers should report kappa, the prevalence, positive agreement, negative agreement, and the relative frequency in each cell (i.e. *a*, *b*, *c *and *d*) to enable the reader to judge the validity of administrative data from multiple aspects.

## Background

Measuring validity, including content, construction, and criterion validity is a fundamental issue for medical research. In epidemiological studies, criterion validity (referred to simply as validity in this paper), is most widely used, and depends heavily on the criterion measurement. Ideally, the criterion should completely reflect the 'truth', and is commonly referred to as the 'gold standard'. Upon implementation of the 'gold standard', statistics of sensitivity, specificity, positive predictive value (PPV) and negative predictive value (NPV) are employed to quantify the validity of a measure that is being examined.

In the 'real' world, particularly in epidemiological studies, a 'gold standard' is rarely available, too difficult, or costly to establish. Therefore researchers often utilize proximate measures of the 'gold standard' as the criterion to assess validity. For example, the validity of medical conditions recorded in hospital discharge administrative data has been assessed by re-abstraction of inpatient charts by health professionals, as well as comparison with patient self-reported data. In these situations when there is no 'gold standard', the kappa statistic is commonly used to assess agreement for estimating "validity".

In 1960, based on the chance-corrected reliability of content analysis[1], Cohen developed the kappa statistic for evaluation of categorical data, which corrects or adjusts for the amount of agreement that can be expected to occur by chance alone[2]. Since its inception, kappa has been widely studied and critiqued (Table 1). One common criticism is that kappa is highly dependent on the prevalence of the condition in the population[3,4]. To overcome this limitation, several alternative methods for agreement have been investigated [5-8]. In 1993, Byrt et al[9] proposed a bias-adjusted and prevalence-adjusted kappa (PABAK) that assumes fifty percent prevalence of the condition, and absence of any bias. PABAK has been employed in many pervious studies for agreement assessment [10-17]. Compared with kappa, PABAK reflects the ideal situation, and ignores the variation of prevalence across the conditions and bias presented in the "real" world. To demonstrate the performance of kappa and PABAK, we assessed the agreement between hospital discharge administrative data and chart review data conditions, considering that the prevalence of a condition varies by the sampling method employed. We analyzed kappa and PABAK in the following three sampling scenarios; 1) random sampling, 2) restricted sampling by conditions, and 3) case-control sampling.

**Table 1:** Literature related to kappa

Author	Main contribution
Cohen[2]	Investigate chance-corrected kappa agreement coefficient and its standard error, techniques for estimation, and hypothesis testing
Cohen[27]	Provide weighted version of kappa for ordinal statistics
Fleiss et al[28]	Investigate asymptotic variance formula for testing kappa and CI of kappa for general *m *× *m *table
Landis et al[29]	Provide arbitrarily the range of kappa value to the degree of agreement
Fleiss[30], Landis et al[31]	Extended kappa for more than 2 raters, and multiple categories
Bloch et al[5]	Investigate intraclass kappa and its CI estimation
Cicchetti et al[3], Feinstein et al[4]	Investigate the effect of bias and prevalence on kappa
Barlow[32]	Investigate kappa statistics included covariates
Oden et al[33]	Extended the kappa for paired-data
Byrt et al[9]	Introduced prevalence-adjusted bias-adjusted kappa
Vach[22]	The effects of prevalence and bias on kappa is negligible

## Methods

### Random sample

A total of 4,008 hospital discharge records were randomly selected from the four adult teaching hospitals in Alberta, Canada among admissions during January 1, 2003 and June 30, 2003. There were at least 1000 records from each hospital.

### Defining conditions in ICD-10 administrative data

Professional trained health record coders read through the patient' medical chart to assign International Classification of Disease 10^th ^version (ICD-10) diagnoses that appropriately described the patient's hospitalization. Each discharge record contained a unique identification number for each admission, a patient chart number, and up to 16 diagnoses. We defined 32 conditions based on ICD-10 codes[18].

### Defining conditions in chart data

Charts of the randomly selected 4008 patients were located using the personal unique identifier and admission date. Two professionally trained reviewers completed a thorough chart review of 4008 patients through examining the chart cover page, discharge summaries, narrative summaries, pathology reports (including autopsy reports), trauma and resuscitation records, admission notes, consultation reports, surgery/operative reports, anesthesia reports, physician daily progress notes, physician orders, diagnostic reports, and transfer notes for evidence of any of the 32 conditions. The process took approximately one hour for each chart.

### Restricted sample

We extracted records with any one of three conditions (i.e. hypertension, myocardial infarction and congestive heart failure) from the ICD-10 administrative data. Among 1126 records that met the criteria, there were 887 records with hypertension, 336 with myocardial infarction and 254 with congestive heart failure.

### Case-control sample

We defined a case-control sample for each of the three specific conditions based on ICD-10 administrative data. The first sample included 887 records with hypertension and 887 randomly selected records among those without hypertension. Thus in total the sample for hypertension contained 1774 records. Using the same method, the second and third sample was generated for myocardial infarction and congestive heart failure. In total there were 672 records for myocardial infarction (336 with myocardial infarction, 336 without) and 508 records for congestive heart failure (254 with congestive heart failure and 254 records without).

### Statistical indices of agreement

We calculated the prevalence of condition, kappa, PABAK statistic, positive agreement, negative agreement, observed agreement, and chance agreement for the condition in each of three samples. The definition of kappa and PABAK is:

$$\begin{array}{cc} \kappa =\frac{I_{o}-I_{e}}{1-I_{e}}, & PABAK=2I_{o}-1 \end{array}$$

Where *I*_*o *_and *I*_*e *_is observed agreement and chance agreement, respectively.

The cross-classification for the results of the condition by two databases and the formulas for calculating agreement statistics can be found in Additional file 1. We calculated statistical values in the three samples, such as kappa. These refer to statistics for samples and do not refer to parameter values for populations although the sample statistics are used to estimate population values. Therefore, the statistical indices used in this paper refer to the statistical value for sample, not for population.

This study was approved by the ethics committee of University of Calgary, Canada

## Results

### Random sample for 32 conditions

**Figure 1:**
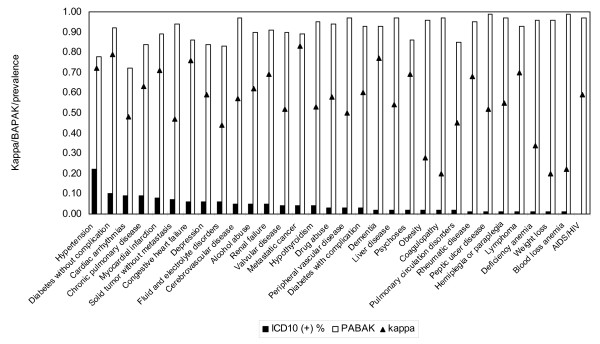
**The comparison of kappa and PABAK with changes of the prevalence of the conditions**.

The statistical indices for agreement for the 32 conditions are presented in table 2. The prevalence of the conditions for ICD-10 administrative data ranged from 0.25% to 22.13%, whereas for chart review, it was from 0.60% to 30.19% among the 32 conditions. The variation for negative agreement was from 0.92 to 1.00, and from 0.21 to 0.84 for positive agreement. The kappa varied from 0.20 to 0.83, whereas for PABAK, it was from 0.72 to 0.99, with the PABAK value greater than kappa for all conditions in the sample. The difference between PABAK and kappa values ranged from 0.06 to 0.77. Hypertension and metastatic cancer had the lowest difference of 0.06, while blood loss anemia and coagulopathy had the most extreme difference of 0.77. The kappa and PABAK with the prevalence of 32 conditions illustrate in the figure 1.

**Table 2:** Prevalence and reliability index between chart abstract and ICD-10 discharge abstract data for 32 conditions

	Chart (+) % *p*_1_	ICD10 (+) % *p*_2_	Chart(+) ICD10(+) %,(*a*)	Chart(-) ICD10(+) %,(*b*)	Chart(+) ICD10(-) %,(*c*)	Chart(-) ICD10(-) %, (*d*)	PABAK	Kappa	Positive agreement	Negative agreement
Hypertension	30.19	22.13	20.61	1.52	9.58	68.29	0.78	0.72	0.79	0.92
Diabetes without complication	11.88	10.18	9.01	1.17	2.87	86.95	0.92	0.79	0.82	0.98
Cardiac arrhythmias	21.80	9.10	8.50	0.60	13.30	77.60	0.72	0.48	0.55	0.92
Chronic pulmonary disease	15.00	8.71	7.91	0.80	7.09	84.21	0.84	0.63	0.67	0.96
Myocardial infarction	12.75	8.38	7.83	0.55	4.92	86.70	0.89	0.71	0.74	0.97
Solid tumor without metastasis	9.51	7.44	4.37	3.07	5.14	87.43	0.84	0.47	0.52	0.96
Congestive heart failure	8.33	6.34	5.71	0.62	2.62	91.04	0.94	0.76	0.78	0.98
Depression	11.90	5.84	5.34	0.50	6.56	87.60	0.86	0.57	0.60	0.96
Fluid and electrolyte disorders	11.05	5.61	4.02	1.60	7.04	87.35	0.83	0.44	0.48	0.95
Renal failure	3.99	4.89	3.14	1.75	0.85	94.26	0.95	0.69	0.71	0.99
Alcohol abuse	7.36	4.59	3.84	0.75	3.52	91.89	0.91	0.62	0.64	0.98
Cerebrovascular disease	8.13	4.54	3.77	0.77	4.37	91.09	0.90	0.57	0.59	0.97
Metastatic cancer	4.42	4.12	3.57	0.55	0.85	95.03	0.97	0.83	0.84	0.99
Hypothyroidism	8.83	3.72	3.47	0.25	5.36	90.92	0.89	0.53	0.55	0.97
Valvular disease	6.96	3.54	2.84	0.70	4.12	92.34	0.90	0.52	0.54	0.97
Drug abuse	4.92	2.82	2.30	0.52	2.62	94.56	0.94	0.58	0.59	0.98
Peripheral vascular disease	4.27	2.82	1.85	0.97	2.42	94.76	0.93	0.51	0.52	0.98
Diabetes with complication	2.74	2.57	1.62	0.95	1.12	96.31	0.96	0.60	0.61	0.99
Dementia	3.32	2.40	2.22	0.17	1.10	96.51	0.97	0.77	0.78	0.99
Liver disease	5.04	2.40	2.05	0.35	2.99	94.61	0.93	0.54	0.55	0.98
Obesity	8.31	1.85	1.55	0.30	6.76	91.39	0.86	0.28	0.30	0.96
Psychoses	2.89	1.82	1.65	0.17	1.25	96.93	0.97	0.69	0.70	0.99
Coagulopathy	7.71	1.80	1.07	0.72	6.64	91.57	0.85	0.20	0.23	0.96
Pulmonary circulation disorders	2.69	1.62	1.00	0.62	1.70	96.68	0.95	0.45	0.46	0.99
Rheumatic disease	2.59	1.42	1.37	0.05	1.22	97.36	0.97	0.68	0.68	0.99
Deficiency anemia	1.90	1.42	0.57	0.85	1.32	97.26	0.96	0.34	0.35	0.99
Hemiplegia or paraplegia	1.55	1.40	0.82	0.57	0.72	97.88	0.97	0.55	0.56	0.99
Peptic ulcer disease	2.52	1.30	1.00	0.30	1.52	97.18	0.96	0.52	0.52	0.99
Weight loss	3.74	0.85	0.47	0.37	3.27	95.88	0.93	0.19	0.21	0.98
Lymphoma	1.02	0.82	0.65	0.17	0.37	98.80	0.99	0.70	0.70	1.00
Blood loss anemia	1.12	0.62	0.20	0.42	0.92	98.45	0.97	0.22	0.23	0.99
AIDS/HIV	0.60	0.25	0.25	0.00	0.35	99.40	0.99	0.59	0.59	1.00

### Restricted sample for select conditions

The prevalence of hypertension, myocardial infarction and congestive heart failure for ICD-10 data increased from 22.13%, 8.38%, 6.34% in the randomly selected sample to 78.77%, 29.84% and 22.56% for restricted sample, respectively (see Table 3). The difference between PABAK and kappa values for hypertension, myocardial infarction and congestive heart failure was 0.09, 0.03 and 0.05, respectively that changed from 0.06, 0.18 and 0.18 for the random sample.

**Table 3:** Prevalence and reliability index between chart abstract and ICD-10 discharge abstract data for 3 select conditions, by sampling method

	Chart (+) *p*_1_, %	ICD10 (+) *p*_2_,%	Chart(+) ICD10(+) (*a*),%	Chart(-) ICD10(+) (*b*),%	Chart(+) ICD10(-) (*c*), %	Chart(-) ICD10(-) (*d*),%	Positive agreement	Negative agreement	PABAK	Kappa	(PABAK-kappa)	Observed agreement (*I*_*o*_)	Chance agreement (*I*_*e*_)
**Random sample**													
Hypertension	30.19	22.13	20.61	1.52	9.58	68.29	0.79	0.92	0.78	0.72	0.06	0.89	0.61
Myocardial infarction	12.75	8.38	7.83	0.55	4.92	86.70	0.74	0.97	0.89	0.71	0.18	0.95	0.81
Congestive heart failure	8.33	6.34	5.71	0.62	2.62	91.04	0.78	0.98	0.94	0.76	0.18	0.97	0.86
**Restricted sample**^#^													
Hypertension	78.51	78.77	73.36	5.42	5.15	16.07	0.93	0.75	0.78	0.69	0.09	0.89	0.66
Myocardial infarction	37.92	29.84	27.89	1.95	10.04	60.12	0.82	0.91	0.76	0.73	0.03	0.88	0.55
Congestive heart failure	25.58	22.56	20.34	2.22	5.24	72.20	0.85	0.95	0.85	0.80	0.05	0.93	0.63
**Case-control sample**^$^													
Hypertension	52.20	50.00	46.56	3.44	5.64	44.36	0.91	0.91	0.82	0.82	0.00	0.91	0.50
Myocardial infarction	49.70	50.00	46.73	3.27	2.98	47.02	0.94	0.94	0.88	0.88	0.00	0.94	0.50
Congestive heart failure	45.47	50.00	45.08	4.92	0.39	49.61	0.94	0.95	0.89	0.89	0.00	0.95	0.50

#: The sample restricted to the condition of hypertension, myocardial infarction, and congestive heart failure

$: Condition chosen first, with random selection of controls without the condition of interest.

### Case-control sample for select conditions

By design the prevalence for these three conditions for ICD-10 administrative data was 50%, both PABAK and kappa values for hypertension, myocardial infarction and congestive heart failure were 0.82, 0.88, and 0.89, resulting in a difference of 0 between these two indices.

## Discussion

We investigated the performance of prevalence unadjusted (i.e. kappa) and adjusted statistical indices (i.e. PABAK) to assess agreement between administrative data and medical chart review data in a randomly selected sample for 32 conditions, as well as a restricted, and case-control sample for three select conditions (hypertension, myocardial infarction and congestive heart failure). Our results indicate that for the same condition the prevalence varies depending on the sampling method, and this variation affects both the kappa and PABAK statistic. We highlight that 1) kappa values varied more widely than PABAK values across the 32 conditions; 2) PABAK should usually not be interpreted as measuring the same agreement parameter as kappa in administrative data, particular for the condition with low prevalence; 3) the gap between these two statistical values for the same condition became narrowed with an increase of its prevalence and disappeared when the prevalence was fixed to 50%.

The sampling method employed has a significant effect on the assessment of validity. Currently, random sample, restricted sample, and case-control sample are popularly used in validity study for administrative data [19-21]. In our study, the kappa value for hypertension is 0.72 for random sample and 0.69 for restricted sample. Previous studies indicate that kappa value is highly dependent on the prevalence of the condition[3,4]. The prevalence of hypertension is 22.13% for the random sample and 78.77% for the restricted sample in this study. Therefore, the difference of kappa value for hypertension might be caused by the different prevalence between random and restricted samples. The sampling method also impacted the PABAK value, with the PABAK value varying by type of sampling method. By definition the PABAK assumes the prevalence is 50% with zero bias[6]; its value only depends on the observed agreement[9]. It reaches to 0.82 for case-control sample when prevalence of hypertension is 50%. These findings are consistent with Vach's[22] report based on the hypothetical sample. In order to overcome the effect of prevalence on kappa value, some researchers advocate using a balance sample and avoiding kappa to assess validity of conditions with low or very high prevalence [3,4,9,23]. A potential reason for the variation in PABAK by sampling methods is due to the change in observed agreement, which was a result of the variation in prevalence estimates by sampling methods. Interpretation of validation study results should therefore consider sampling effect and the disease prevalence.

Kappa is sensitive to the prevalence of a condition defined from administrative data. Agresti's study[24] investigated the influence of prevalence on kappa, and compared the kappa values from populations with different prevalence. Vach's[22] study indicated the kappa is highest when prevalence equals 50%. In reality, nearly all diseases have prevalence lower than 50% in populations. Case-control study design (1:1 matching) automatically maximizes kappa value. In our study, kappa for hypertension in the case-control sample of 0.82 was higher than that in the random sample of 0.72. Therefore, caution should be used in interpreting kappa for case-control studies.

In this study, we compared performance of kappa and PABAK in measuring agreement between administrative data and chart review data but were unable to determine which statistic measures agreement more accurately and reliably. The reason is that we could not establish parameter of 'true' validity of administrative data. However, assessment of these two statistics through administrative data coding practice (i.e. face-validity) demonstrates that PABAK values should usually not be interpreted as measuring the same agreement parameter as kappa, particular for the condition with low prevalence. In our random sample, PABAK value ranged from 0.72 to 0.99 among 32 conditions, indicating a high degree of "validity" for administrative data in recording these conditions. The high PABAK value for obesity and weight loss are examples (0.96 for both conditions) that question the PABAK validity. Administrative data coding guideline and practice[25] instructs coders not to code these conditions even if they are documented in charts because they may not affect length of stay, healthcare or therapeutic treatment. Additionally, coders may intentionally not code these conditions due to the limited amount of time given to code each chart. Therefore, these two conditions generally have very poor validity in administrative data. The fact is revealed by the very poor positive agreement (0.30 for obesity and 0.21 for weight loss, respectively) and kappa value (0.20 and 0.28). PABAK assumes the bias is absent and the prevalence is 50%. However, when bias presented and the prevalence departed from 50%, the PABAK value and kappa value are inconsistence.

Bias also has an effect on the kappa values. Bias is the extent to which there is disagreement on the proportion of positive (or negative) cases and is reflected in a difference between *b *and *c *(see Additional file 1)[26]. The kappa value is higher for a large bias, while the kappa value is lower for lower or absent bias[9]. In our study, the value of *b *and *c *changed for the same condition in the different samples. For myocardial infarction, the value of *b *and *c *was 0.55% and 4.92% in the random sample, respectively, whereas it was 1.95% and 10.04% in the restricted sample. Furthermore, the prevalence of the condition also changed from 8.38% for random sample, and 29.84% for restricted sample, respectively. According to the definition of kappa, it is obvious that the changes of prevalence have the effect on kappa. For the restricted sample, both the difference of *b *and *c*, and the prevalence of myocardial infarction are higher compared to the random sample. This partly explains why the kappa value is higher in the restricted sample than those in the random sample.

Our study at least has two limitations. Firstly, we cannot capture the 'true' value of the kappa for the conditions in the administrative data in our study. Therefore, the difference between the 'true' value and estimated value of kappa, and their changes due to the variation of prevalence, cannot be evaluated. Secondly, we employed chart data extracted by reviewers as a 'gold standard' to assess the validity of ICD-10 data. Such a criterion standard depends on the quality of charts.

## Conclusion

Our study indicates the prevalence of conditions varies depending on the sampling method employed, and these changes have an effect on kappa and PABAK. Although PABAK theoretically adjusts for prevalence, this statistic may be high to evaluate the agreements between two data sources, and may result in misleading conclusion. Although no single agreement statistic can capture the desired information, we encourage researchers to report kappa, the prevalence, positive agreement, negative agreement, and the relative frequency of in each cell (i.e. *a*, *b*, *c *and *d*) to permit readers to judge the validity of administrative data from multiple aspects.

## Competing interests

The authors declare that they have no competing interests.

## Authors' contributions

GC involved in study design, performed statistical analysis, interpreted the results and drafted the manuscript. PF and BH participated in the study design and interpretation of the results. RB involved in interpretation of the results and participated in preparation the manuscripts. HQ conceived the study, participated in its design and interpreted results. All authors read and approved the final manuscript.

## Pre-publication history

The pre-publication history for this paper can be accessed here:

http://www.biomedcentral.com/1471-2288/9/5/prepub
